# Microbial Communities in Long-Term, Water-Flooded Petroleum Reservoirs with Different *in situ* Temperatures in the Huabei Oilfield, China

**DOI:** 10.1371/journal.pone.0033535

**Published:** 2012-03-14

**Authors:** Yue-Qin Tang, Yan Li, Jie-Yu Zhao, Chang-Qiao Chi, Li-Xin Huang, Han-Ping Dong, Xiao-Lei Wu

**Affiliations:** 1 Department of Energy and Resources Engineering, College of Engineering, Peking University, Beijing, People's Republic of China; 2 College of Architecture and Environment, Sichuan University, Chengdu, Sichuan, People's Republic of China; 3 Research Institute of Petroleum Exploration and Development (Langfang), China National Petroleum Corporation (CNPC), Beijing, People's Republic of China; University of Nottingham, United Kingdom

## Abstract

The distribution of microbial communities in the Menggulin (MGL) and Ba19 blocks in the Huabei Oilfield, China, were studied based on 16S rRNA gene analysis. The dominant microbes showed obvious block-specific characteristics, and the two blocks had substantially different bacterial and archaeal communities. In the moderate-temperature MGL block, the bacteria were mainly *Epsilonproteobacteria* and *Alphaproteobacteria*, and the archaea were methanogens belonging to *Methanolinea*, *Methanothermobacter*, *Methanosaeta*, and *Methanocella*. However, in the high-temperature Ba19 block, the predominant bacteria were *Gammaproteobacteria*, and the predominant archaea were *Methanothermobacter* and *Methanosaeta*. In spite of shared taxa in the blocks, differences among wells in the same block were obvious, especially for bacterial communities in the MGL block. Compared to the bacterial communities, the archaeal communities were much more conserved within blocks and were not affected by the variation in the bacterial communities.

## Introduction

Due to increasing energy demand and depletion of oil reserves, the development of alternative enhanced oil recovery (EOR) techniques in place of water flooding is gaining increased attention to enhance oil productivity and recovery efficiency. Among these so-called tertiary recovery techniques, microbial enhanced oil recovery (MEOR) is considered to be much more economically feasible due to its low energy consumption, low environmental impact, and cost-effectiveness [1]. The MEOR technique can be classified as two types: single-well huff-and-puff and “microbial flooding”. In both cases, microbial communities are stimulated to degrade petroleum constituents, producing biosurfactants, gases, and other by-products that increase the crude oil fluidity. Therefore, understanding the composition of the microbial community and the stimulation strategies is the crucial starting point for the whole process.

In the past two decades, use of culture-dependent and culture-independent approaches to examine microbial communities in oil reservoirs has revealed physiologically and phylogenetically diverse microorganisms inhabiting the reservoirs [1]–[10]. Microbial communities differ among oil reservoirs. Most studies have investigated the microbial community from only one or at most three oil wells [4], [5], [9], [11]. It is not clear if the microbial community from one to three wells can represent an entire oil block, much less an entire oil field. Understanding the microbial community distribution in a block is crucial for the “microbial flooding” oil recovery process. The lack of information hinders the development and application of the MEOR process. In addition, even for the single-well huff-and-puff technique, the microbial community may be different upon change of the working layer. In fact, the microbial community can be influenced by characteristics of the reservoir, the working layer, the oil composition, as well as the injection water.

Water flooding is an oil recovery process currently employed worldwide. Especially in China, water flooding has been used for decades at most oil reservoirs. Fresh water from lakes, rivers, and/or aquifers is injected into oil reservoirs to increase the reservoir pressure for enhanced oil recovery. After extraction from the production wells, the water is treated with an oil-water-sand three-phase separation process, and sometimes with distillation to remove harmful microorganisms such as sulfate-reducing bacteria. Most of the treated water is re-injected into the oil reservoirs via injection wells and continuously recycled. Fresh water is occasionally added into the injection water to compensate for water lost in the course of oil recovery, including water lost by retention in the oil reservoir to replace the volume of oil that is extracted. It is therefore reasonable that the microbial community in the injection water will also influence the community in the reservoir. However, the extent of influence remains unclear although two recent studies examined microbial communities in injection water [9], [12].

For more insight into the microbial community in oil fields, we conducted a systematic investigation in oil reservoirs with different characteristics in China, including the Huabei Oilfield, Shengli Oilfield, Daqing Oilfield, Xinjiang Oilfield, Qinghai Oilfield, and Liaohe Oilfield. As part of the project, the bacterial and archaeal communities of two typical blocks of the Huabei Oilfield with a total of 13 oil wells and 2 injection water stations were investigated using clone library and T-RFLP analysis. The Huabei Oilfield subproject is the focus of this report.

## Materials and Methods

### Sampling sites and sample collection

We obtained permission from the Research Institute of Petroleum Exploration and Development (China National Petroleum Corporation (CNPC)) for observation and field studies in Menggulin (MGL) oilfield and Baolige oilfield. Samples were collected from a Menggulin (MGL) sandstone block in the MGL oilfield as well as from the Ba19 fault block in the Baolige oilfield (Huabei Oil Field Ltd.) located in the central part of Inner Mongolia, China. The distance between the two blocks was approximately 50 km. MGL oilfield has been water flooded since 1989. The average water content at present is approximately 95%. Baolige oilfield has been water flooded since 2001, and the average water content at present is approximately 78%. The injection water was recycled after the oil-water separation of production water from the oil wells. The characteristics of the two blocks are shown in Table 1. The well bottom temperatures were 37°C and 58.4°C for MGL and Baolige oilfields, respectively.

**Table 1:** Characteristics of the Menggulin (MGL) and Ba19 blocks in Huabei Oilfield, China.

Parameters	Block MGL	Block Ba19
Oil Reservoir		
Depth (m)	∼806	∼1380
Temperature (°C)	∼37.0	∼58.4
Pressure (MPa)	∼7.46	∼13.8
Porosity	∼23.6	∼18.5
Air permeability	∼675.3	∼691.0
Crude Oil		
Viscosity *in situ* (mPa·s)	∼179.1	∼13.68
Element content (%)	S: 0.18; N: 0.38	S: 0.18; N: 0.17
Group composition (%)	saturates:38.94; aromatics:17.77;resins:24.47 asphaltenes: 18.83	saturates: 44.25; aromatics: 27.86;resins: 16.48; asphaltenes: 11.42
Geochemical parameter	Max-peak: nC21;ΣC21^−^/ΣC22^+^: 1.24;C(21+22)/C(28+29): 1.71;Pr/Ph: 0.89; Pr/nC17: 0.58;Ph/nC18: 0.72; CPI: 1.08; OEP:1.16	Max-peak: nC21 or nC23; ΣC21^−^/ΣC22^+^: 1.05; C(21+22)/C(28+29): 1.74;Pr/Ph: 0.71; Pr/nC17: 0.59;Ph/nC18: 0.88; CPI: 1.12; OEP: 1.17
Production water		
Type	NaHCO_3_	NaHCO_3_
pH	8.2–8.6	8.4–9.2
Average salinity (mg/l)	∼2224	∼4015
Average S-TOC (mg/l)	∼93	∼146
Injection water		
pH	8.6–8.9	8.3–8.5
Average salinity (mg/l)	∼1965	∼3478
Average S-TOC (mg/l)	∼51	∼38

**Figure 1:**
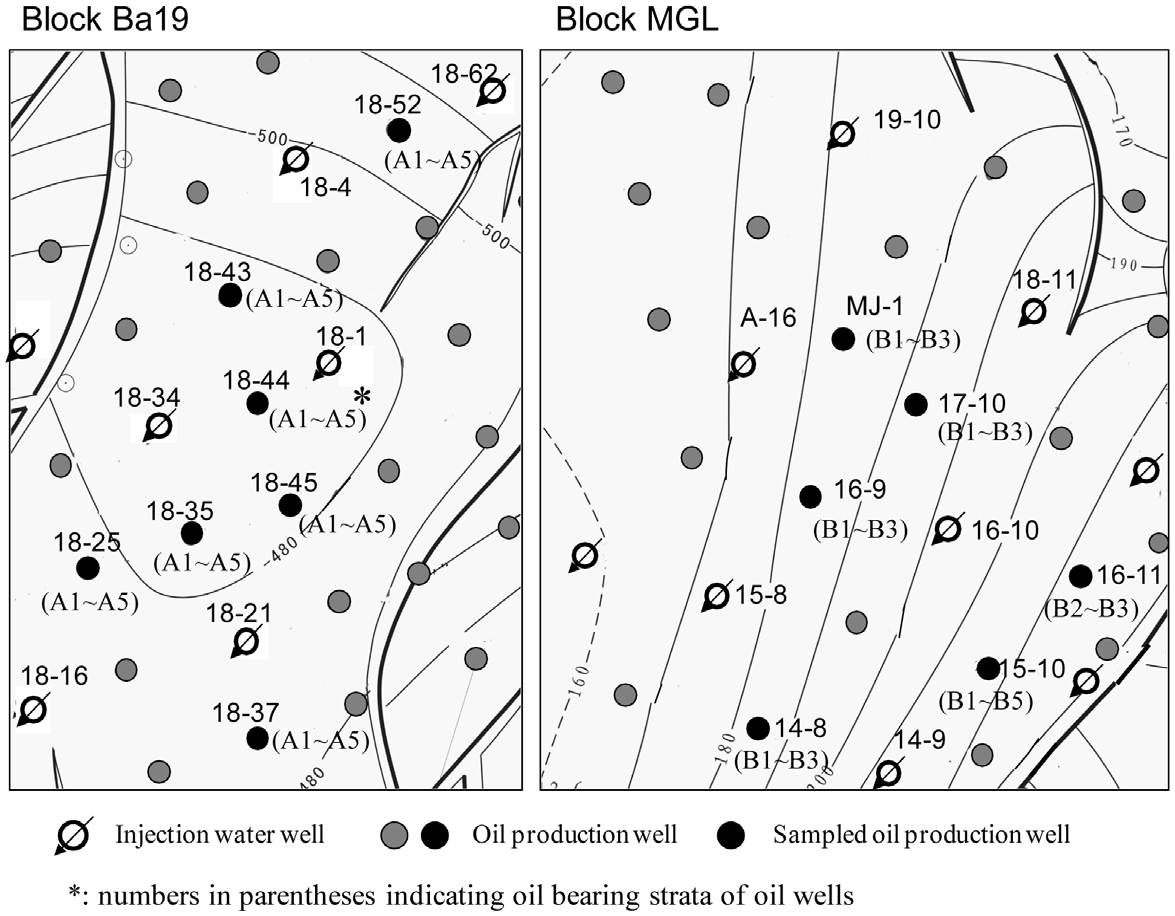
Distribution of injection water wells and oil production wells in the MGL and Ba19 blocks.

The injection water and production water were collected in December, 2009 from the water stations and from different wellheads, respectively, in both blocks. In total, six oil wells from the MGL block and seven oil wells from the Ba19 block were investigated. The injection wells and oil production wells located were near each other; the distances between wells ranged from 0.2 km to 0.5 km. However, differences occurred in oil-bearing strata among wells, especially among wells in the MGL block (Fig. 1). The injection water and production water (mixture of crude oil and water) samples were collected by fully filling 5 liter plastic sampling bottles. The samples were transported to the laboratory at ambient (approximately 5°C) temperature. In the laboratory, the production water was separated and the floating oil was collected for oil composition analysis. The remaining water phase was filtered through 0.22 µm membrane filters. The filtrates were analyzed to determine the concentrations of ions. The cells trapped on the membrane filter were used to extract DNA for microbial community analysis.

### Chemical analysis

The concentrations of cations and anions in injection water and water from oil wells were analyzed using an ion chromatograph (PLA-1000, Shimadzu, Kyoto) with a Shim-pack IC-A3 column for cation analysis and a Shim-pack IC-C3 column for anion analysis. The concentrations of total organic carbon (TOC) were analyzed using a TOC auto analyzer (TOC-V; Shimadzu, Kyoto). Crude oil samples from the same block were mixed and dehydrated before analysis. The sulfur (S) and nitrogen (N) content in oil was analyzed using a multi EA 3100 elemental analyzer (Jena, Germany). The contents of saturates, aromatics, resins and asphaltenes in oil were determined by fractionating with liquid-solid chromatography [13]. The distribution of alkanes in oil was analyzed according to the Chinese Standard SY/T 5779-2008 using gas chromatography (Agilent 7890A, USA) with an HP-5MS column (60 m×0.25 mm×0.25 µm) and nitrogen as the carrier gas at a flow rate of 1 mL/min. The temperature of the injector and the detector was 300°C. The temperature program was: 40°C for 10 min, increase by 4°C/min to 300°C, hold for 30 min.

### DNA extraction and PCR amplification of 16S rRNA genes

DNA was extracted from samples using the FastDNA® Spin Kit for Soil (MP Biomedicals, Cleveland, USA). Bacterial and archaeal 16S rRNA genes were amplified with primer sets Eu27F (5′-AGA GTT TGA TCC TGG CTC AG-3′) and 1492R (5′-GGT TAC CTT GTT ACG ACT T-3′), and Ar109F (5′-ACA GCT CAG TAA CAC GT-3′) and Ar915R (5′-GTG CTC CCC CGC CAA TTC CT-3′) [14], [15], respectively. PCR mixtures (50 µl) contained 5 µl of 10× PCR buffer with 15 mmol/l MgCl_2_ (Takara, Japan), 200 µmol/l of each dNTP (Takara, Dalian, China), 10 pmol of each primer (Applied Biosystems, Foster City, CA, USA), 1.5 units of Taq DNA polymerase (Takara), and 1 µl of DNA template. PCR was performed in a PTC-200 thermocycler (Bio-Rad, Germany). The PCR conditions were: 94°C for 5 min, followed by 30 cycles of denaturation at 94°C for 1 min, annealing at 50°C for 45 s, and extension at 72°C for 1.5 min (for archaea) or 2 min (for bacteria). The 6-carboxyfluorescein (6-FAM)-labeled Eu27F and Ar915R primers were used to amplify the 16S rRNA gene fragment for bacterial and archaeal terminal restriction fragment length polymorphism (T-RFLP) analyses, respectively. PCR products were purified with the QIAquick PCR Purification Kit (Qiagen, Shanghai, China).

### T-RFLP analysis

All samples collected from both blocks (two injection waters and production waters from 13 oil wells) were subjected to both bacterial and archaeal T-RFLP analyses. The analysis was carried out as previously described [16]. Purified DNA amplicons were digested, desalted, mixed with formamide and a DNA fragment length internal standard, and denatured. The “Genescan” analysis was then conducted in a capillary electrophoresis system (ABI 3130 Genetic Analyzer, Applied Biosystems) according to the manufacturer's instruction. Fragment separation data were collected with ABI 3130 Collection (version 2.7) and GeneMapper Analysis Software (version 3.7). The relative peak area of every T-RF was calculated by dividing the individual T-RF peak area by the total area of all the peaks. Relative abundances of T-RF peaks in each sample were used as input for STATISTICA 6.0 software (Statsoft, OK, US), and cluster analysis was carried out based on the calculation of Euclidean distances. T-RFLP analysis of representative clones in each clone library (see below) was performed simultaneously with analysis of the community DNA, and then the taxonomic assignment of T-RF peaks of community samples was determined.

### Clone library construction, screening, sequencing, and phylogenetic and statistical analyses

Among the 15 samples, the 2 injection water samples and 5 production water samples from oil wells, namely M16-9, M17-10, B18-43, B18-44, and B18-45 (Table 2), were selected for clone library analysis of bacterial and archaeal communities. Using a procedure described previously [16], the clones in each library were randomly picked and classified into different phylotypic groups according to the patterns of RFLP digestion with the enzymes *Rsa*I and *Msp*I (for bacterial clones) or *Taq*I (for archaeal clones) (New England BioLabs, MA, USA) as described by Yu et al. [16]. Representative clones from different phylotype groups were randomly selected and sequenced. After performing a chimera check, the sequences with greater than 97% similarity were grouped as one operational taxonomic unit (OTU). Alignments of the representative OTU sequences and their reference sequences were made using ClustalX [17], and phylogenetic trees were constructed by the neighbor-joining method with the Molecular Evolutionary Genetics Analysis (MEGA) software 4.0 [18].

**Table 2:** Richness and diversity of 16S rRNA gene sequences in clone libraries.

Library	Clone No.	OTU No.	Chao-1	Shannon	Simpson	Coverage	Boneh
*Bacteria* library							
MW-B	173	48	100.14	3.35	0.05	0.85	3.52
M16-9-B	92	12	29.00	1.36	0.37	0.89	1.99
M17-10-B	180	18	29.50	1.78	0.31	0.96	0.96
Ba19W-B	78	14	17.75	1.55	0.39	0.92	1.75
B18-43-B	131	4	4.00	0.38	0.83	0.99	0.18
B18-44-B	137	3	3.00	0.73	0.49	0.99	0.13
B18-45-B	92	5	8.00	0.58	0.70	0.97	0.53
*Archaea* library							
MW-A	87	10	10.20	1.66	0.24	0.98	1.00
M16-9-A	87	7	7.00	1.55	0.26	1.00	0.00
M17-10-A	76	13	31.00	1.41	0.41	0.88	1.95
Ba19W-A	86	7	8.00	1.00	0.46	0.97	0.81
B18-43-A	90	2	2.00	0.39	0.77	1.00	0.00
B18-44-A	87	4	4.00	0.62	0.71	1.00	0.00
B18-45-A	95	6	6.00	1.20	0.42	0.99	0.18

Sequences from each library were analyzed using the mothur program [19] to estimate the species richness and diversity of each library and the distribution of OTUs among different libraries. The Chao1 estimator for species richness and the Shannon and Simpson indices for diversity were calculated with 95% confidence intervals and sequence similarity cutoff set at 3%. The Good's coverage index of a given clone library describes the extent to which the sampled sequences in a library represent the total population. The Boneh index was calculated with sampling size set at 50.

### Nucleotide sequence accession numbers

The nucleotide sequences determined in this study have been deposited in the GenBank database under accession numbers JQ088327 to JQ088469.

## Results

### Physicochemical characteristics of the Menggulin (MGL) sandstone and Ba19 fault blocks

The surface distance between the two blocks was approximately 50 km, however, the physicochemical characteristics including the bottom hole temperatures, the depths, and the pressures of the two oil reservoirs were relatively different (Table 1). Block Ba19 had a high-temperature (58.5°C) while block MGL had a much lower temperature of 37°C. Crude oil in the MGL block had a lower content of saturates and aromatics, suggesting stronger biodegradation. However, differences in geochemical parameters between crude oil samples from the two blocks were not obvious, indicating similar oil formation characteristics and maturity (Table 1). The nitrogen and sulfur content of crude oil from the two blocks was similar and relatively low (Table 1). Production water of both blocks was a bicarbonated sodic type with total salinity of approximately 2,224 mg/l and 4,015 mg/l for the MGL and Ba19 blocks, respectively. The cation, anion, and TOC concentrations among the production waters in each block were similar (data not shown). All water samples had alkaline pH values at atmospheric pressure, with higher values from the Ba19 block (Table 1). Compared to production water, injection water had lower average salinity and TOC. However, the injection water from the MGL block had a higher pH than production water from the same block, while the opposite pattern occurred for the Ba19 block. In addition, the production wells in the MGL block had different oil-bearing strata ranging from two to five layers, whereas the production wells in the Ba19 block shared same five layers (Fig. 1).

### Frequencies of the 16S rRNA genes in the two blocks

**Figure 2:**
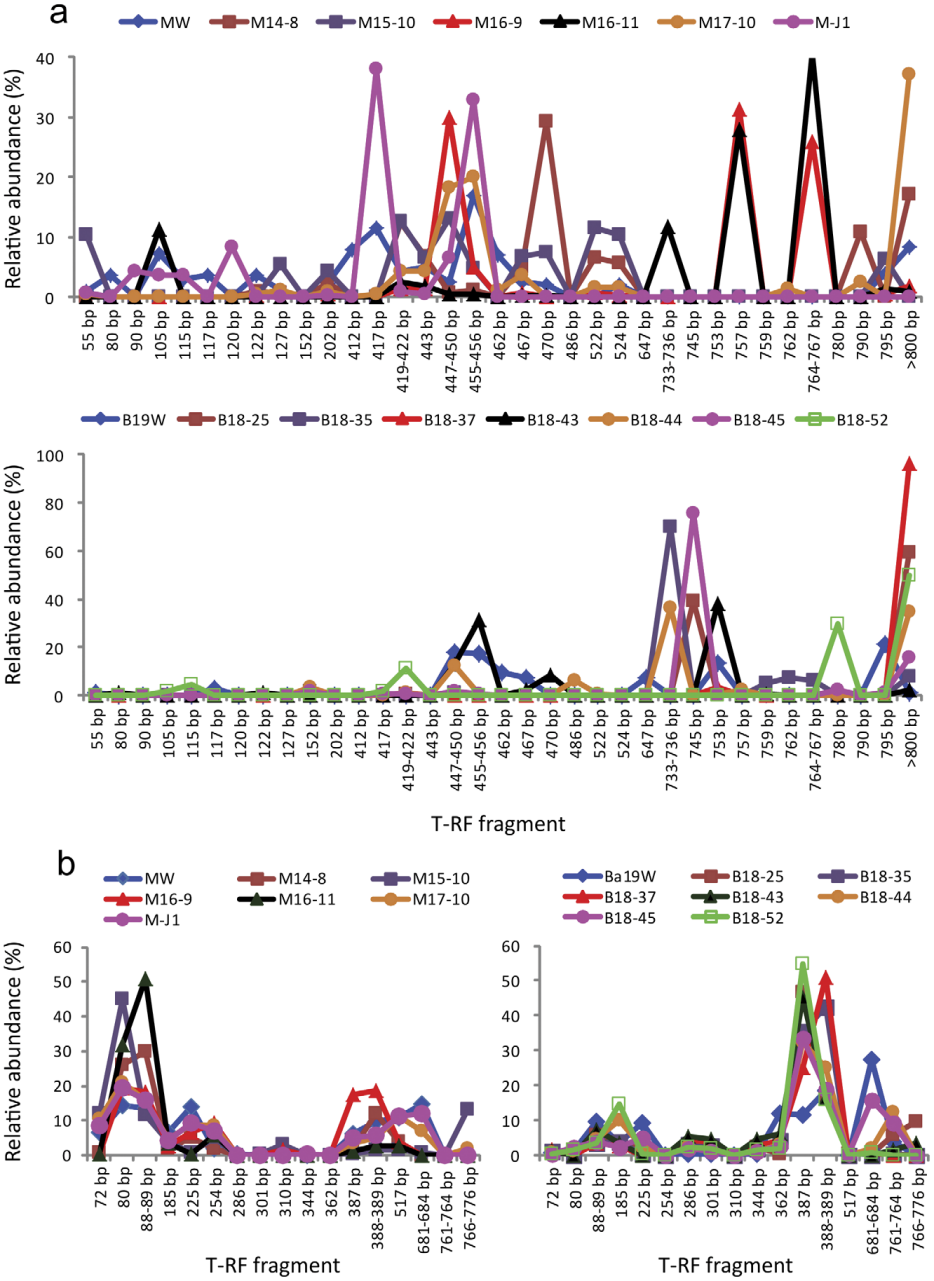
Distribution T-RF peaks of injection waters and production waters from oil wells. Bacterial (a) and archaeal (b) T-RF peaks in the MGL and Ba19 blocks.

T-RFLP analysis of bacterial communities (Fig. 2a) in the 15 samples revealed that two injection waters, MW and Ba19W, had the highest diversity (the most T-RFs) with 20 and 12 T-RFs, respectively. In comparison, there were 11–16 and 4–10 T-RFs in production waters from the MGL and Ba19 blocks, respectively (Fig. 2a). Seventeen T-RFs from MW and eight T-RFs from Ba19W were found in some or all production waters in each block. Eight and 15 T-RFs retrieved from different production waters could not be detected respectively in the MW and Ba19W injection water samples, suggesting the existence of interception and/or inhibition of some bacteria by the reservoirs. Among the production waters in the MGL block, only four common T-RFs were found in all six oil wells. In the Ba19 block, T-RFs 447–450 bp were found in 6 of the production water samples. In contrast, many more archaeal T-RFs were common among the samples inside the MGL block or the Ba19 block (Fig. 2b).

**Figure 3:**
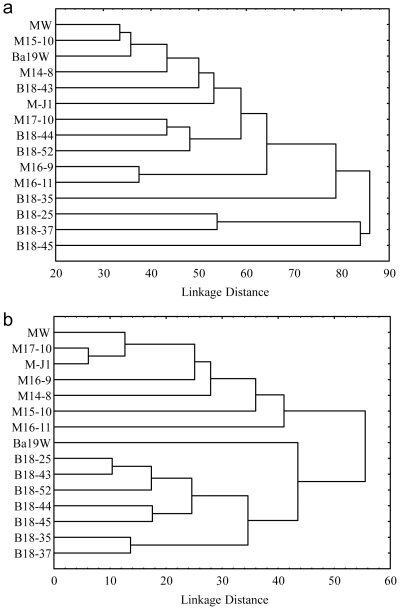
Cluster analysis of microbial communities among samples from the MGL and Ba19 blocks. T-RFLP analysis data was used: bacterial community (a) and archaeal community (b). Relative abundances of each T-RF peak of each sample was used as input for STATISTICA 6.0 software (Statsoft, OK, US).

Cluster analysis of the T-RFLP patterns also revealed the difference in bacterial and archaeal communities between the two blocks, between injection water and production waters, and among production waters (Fig. 3a, 3b). The two injection water samples clustered together, demonstrating the relative similarity of these two communities. Although the production water samples M16-9 and M16-11 clustered and B18-25 and B18-37 clustered, production water communities were generally not block-specific. In contrast, the archaeal communities were clearly block-specific. Again, the injected water communities were readily separated from production water communities. Interestingly, the injection water of the Ba19 block was the out-group in the archaeal tree, while the injection water of the MGL block was the in-group.

### Microbial communities in the two blocks

The bacterial and archaeal 16S rRNA gene clone libraries were constructed with the DNAs from the two injection water samples (MW and Ba19W), two production water samples from the MGL block (M16-9 and M17-10), and three production water samples from the Ba19 block (B18-43, B18-44 and B18-45). The samples were selected because they had a higher number of T-RFs and unique T-RFs. In total, 883 bacterial and 608 archaeal clones were sequenced and assigned to 104 bacterial and 49 archaeal OTUs. The clone libraries for the MGL block contained 12 to 48 bacterial OTUs and 7 to 13 archaeal OTUs (Table 2). The libraries for the Ba19 block contain 3 to 14 bacterial OTUs and 2 to 7 archaeal OTUs. The Good's coverage values were over 85% for the 6 libraries for the MGL block and 92% for the 8 libraries for the Ba19 block (Table 2) indicating the clone sampling approached saturation (as also indicated by the rarefaction analysis shown in Fig. S1 a, b, c). Additionally, the Boneh index values of 0 to 3.52 (for MGL block) and 0 to 1.75 (for Ba19 block) also suggested that the sampled clones adequately represented the microbial communities in the samples. Similar to the results of the T-RFLP analysis, the injection water bacterial communities showed a much higher diversity and richness than those of production waters, indicated by the OTUs obtained, the Chao-1 estimator, as well as Simpson and Shannon indices (Table 2).

#### Bacterial communities in the two blocks

**Figure 4:**
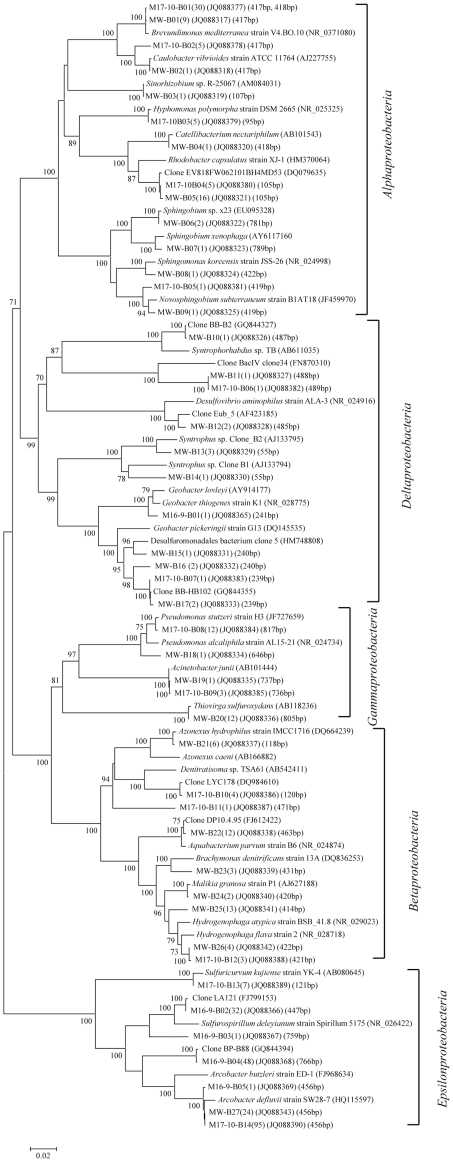
Phylogenetic tree of bacterial clones in the phylum *Proteobacteria* from the MGL block. The tree was constructed by the Neighbor-Joining method using partial sequences of 16S rRNA gene. Numbers of clones with identical sequences and T-RFs of clones are shown in parentheses. The bar represents two substitutions per 100 nucleotide positions. Bootstrap probabilities >70% are indicated at the branch nodes. The DDBJ/EMBL/GenBank accession numbers for reference strains and clones obtained in this study are shown in parentheses.

**Figure 5:**
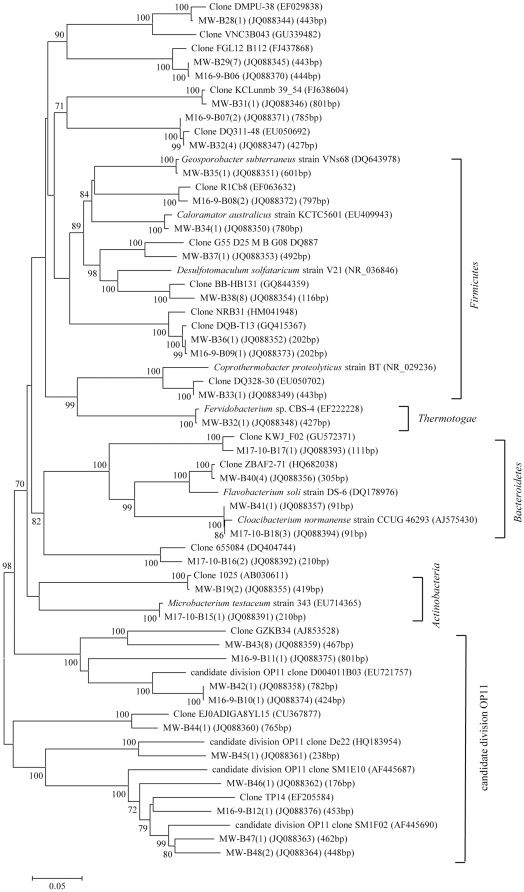
Phylogenetic tree of bacterial clones affiliated with phyla except for *Proteobacteria* from the MGL block. The descriptions are the same as that of Fig. 4.

**Figure 6:**
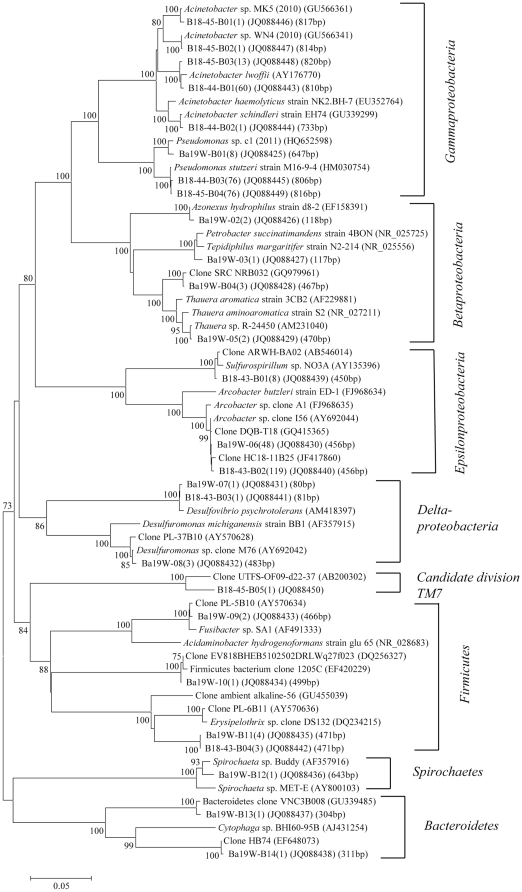
Phylogenetic tree of bacterial clones from the Ba19 block. The descriptions are the same as that of Fig. 4.

The classification and phylogenetic analysis of bacterial clones were shown in Table 3, Fig. 4, Fig. 5 and Fig. 6. In all 7 bacterial clone libraries for the two blocks, bacterial clones assigned to the phylum *Proteobacteria* predominated, accounting for 71.7%, 90.2%, 96.1%, 87.2%, 97.7%, 100%, and 98.9% of total clones in the MW-B, M16-9-B, M17-10-B, Ba19W-B, B18-43-B, B18-44-B, and B18-45-B libraries, respectively (Table 3). The remaining clones were *Firmicutes*, *Bacteroidetes*, *Actinobacteria*, *Thermotogae*, and candidate division OP11 for the MGL block and *Firmicutes*, *Bacteroidetes*, *Spirochaetes*, and candidate division TM7 for the Ba19 block (Table 3). These phyla were minor components of the bacterial community with each having a relative abundance less than 10.0%. Only clones related to *Deltaproteobacteria* and *Epsilonproteobacteria* were common in all MGL bacterial libraries. There was no common bacterial class detected in the Ba19 libraries.

**Table 3:** Classification of bacterial and archaeal clones from the MGL and Ba19 blocks in Huabei Oilfield, China.

Taxon	Block MGL			Block Ba19			
	MW	M16-9	M17-10	Ba19W	B18-43	B18-44	B18-45
	OTU(Clone)	OTU(Clone)	OTU(Clone)	OTU(Clone)	OTU(Clone)	OTU(Clone)	OTU(Clone)
*Bacteria*	48(173)	12(92)	18(180)	14(78)	4(131)	3 (137)	5(92)
*Proteobacteria*	27(124)	5(83)	14(173)	8(68)	3(128)	3 (137)	4(91)
*Alphaproteobacteria*	9(33)	-	5(46)	-	-	-	-
*Betaproteobacteria*	6(40)	-	3(8)	4(8)	-	-	-
*Gammaproteobacteria*	3(14)	-	2(15)	1(8)	-	3 (137)	4(91)
*Deltaproteobacteria*	8(13)	1(1)	2(2)	2(4)	1(1)	-	-
*Epsilonproteobacteria*	1(24)	4(82)	2(102)	1(48)	2(127)	-	-
*Firmicutes*	6(13)	2(3)	-	3(7)	1(3)	-	-
*Bacteroidetes*	2(5)	-	2(4)	2(2)	-	-	-
*Actinobacteria*	1(2)	-	1(1)	-	-	-	-
*Thermotogae*	1(1)	-	-	-	-	-	-
*Spirochaetes*	-	-	-	1(1)	-	-	-
candidate division OP11	7(15)	3(3)	-	-	-	-	-
candidate division TM7	-	-	-	-	-	-	1(1)
Unclassified	4(13)	2(3)	1(2)	-	-	-	-
*Archaea*	10(87)	7(87)	13(76)	7(86)	2(90)	4(87)	6(95)
*Euryarchaeota*							
*Methanosaeta*	3(22)	2(38)	3(4)	2(3)	-	1(4)	2(12)
*Methanosarcina*	-	-	-	1(1)	-	-	-
*Methanomethylovorans*	1(18)	-	1(1)	1(55)	-		1(12)
*Methanolobus*	1(6)	-	1(1)	-	-	-	-
*Methanocella*	1(1)	1(22)	1(11)	-	-	-	-
*Methanoculleus*	-	1(2)	2(2)	-	-	-	-
*Methanospirillum*	1(2)	-	-	-	-	-	-
*Methanolinea*	1(34)	1(11)	1(47)	-	-	-	-
*Methanothermobacter*	1(2)	-	1(1)	2(26)	1(78)	2(77)	1(59)
*Methanobacterium*	1(2)	-	2(8)	-	-	-	-
*Alchaeoglobales*	-	-	-	1(1)	-	1(6)	1(11)
*Crenarchaeota*							
*Thermoprotei*	-	1(9)	1(1)	-	1(12)	-	-
*Thaumarchaeota*							
*Ntrososphaeralesi*	-	-	-	-	-	-	1(1)
Unclassified	-	1(5)	-	-	-	-	-

*Alphaproteobacteria* were only detected in MW-B and M17-10-B libraries, with 9 OTUs (33 clones, 19.1% of total) from MW-B and 5 OTUs (46 clones, 25.6% of total) from M17-10-B. Among them, OTUs MW-B05 (16 clones with a T-RF of 105 bp) and M17-10-B04 (5 clones with a T-RF of 105 bp) did not have closely related cultured species but showed 99% sequence similarity to clone EV818FW06210BH4MD53 (DQ079635) retrieved from a deep terrestrial subsurface fluid-filled fracture. OTUs MW-B01 (9 clones with a T-RF of 417 bp) and M17-10-B01 (30 clones with T-RFs of 417–418 bp) had 99% sequence similarity with *Brevundimonas mediterranea*, which can reduce nitrate using many kinds of carbohydrates [20]. The remaining *Alphaproteobacteria* OTUs detected in MW-B were closely related to *Caulobacter* species (1 clone with a T-RF of 417 bp), *Sinorhizobium* (1 clone with a T-RF of 107 bp), *Catellibacterium* (1 clone with a T-RF of 418 bp), *Sphingobium* (2 clones with a T-RF of 781 bp), *Sphingomonas* (1 clone with a T-RF of 789 bp), and *Novosphingobium* (1 clone with a T-RF of 419 bp). The remaining OTUs from M17-10-B were closely related to *Caulobacter* species (5 clones with a T-RF of 417 bp), *Hyphomonas* (5 clones with a T-RF of 95 bp), and *Novosphingobium* (1 clone with a T-RF of 419 bp) (Fig. 4). Among these bacteria, *Sphingobium*, *Sphingomonas*, and *Novosphingobium* are reported to be able to degrade aromatic compounds [21]–[23].

*Betaproteobacteria* were only detected in MW-B, M17-10-B, and Ba19W-B libraries, with 6 OTUs (40 clones), 3 OTUs (8 clones), and 4 OTUs (8 clones), respectively (Fig. 4, Fig. 6). Among them, 2 OTUs (17 clones with T-RFs of 414 bp and 422 bp) from MW-B and 1 OTU (3 clones with a T-RF of 421 bp) from M17-10-B were closely related to *Hydrogenophaga* species, which are hydrogen-oxidizing bacteria [24]. Other OTUs from MW-B were closely related to *Azonexus* species (6 clones with a T-RF of 118 bp), *Aquabacterium* (12 clones with a T-RF of 463 bp), *Brachymonas* (3 clones with a T-RF of 431 bp), and *Malikia* (2 clones with a T-RF of 420 bp), which are reported to be able to reduce nitrate [25], [26]. Two OTUs (5 clones with T-RFs of 120 bp and 471 bp) of M17-10-B were not closely related to any known species, but one of them (4 clones) was 99% similar to clone LYC178 (DQ984610) obtained from an oil contaminated soil. In the Ba19W-B library, 4 OTUs (8 clones) were closely related to *Azonexus* species (2 clones with a T-RF of 118 bp), *Petrobacter* (1 clone with a T-RF of 117 bp), and *Thauera* (5 clones with T-RFs of 467 bp and 470 bp), which are reported to be able to reduce nitrate as well as degrade aromatic compounds [27]–[29].

*Gammaproteobacteria* were detected in MW-B, M17-10-B, Ba19W-B, B18-44-B, and B18-45-B libraries, with 3 OTUs (14 clones), 2 OTUs (15 clones), 1 OTU (8 clones), 3 OTUs (137 clones, 100% of the total clones), and 4 OTUs (91 clones, 98.9% of the total clones), respectively. Among them, OTU MW-B20 (12 clones with a T-RF of 805 bp) had 99% sequence similarity to *Thiovirga sulfuroxydans*, which can oxidize sulfur under microaerophilic conditions [30]. Other MGL and Ba19 OTUs were closely related to *Pseudomonas* (173 clones with T-RFs of 646–647 bp, 806 bp, 816–817 bp) and *Acinetobacter* (80 clones with T-RFs of 733 bp, 736–737 bp, 810 bp, 814–817 bp, 820 bp) species, which are able to degrade petroleum compounds [31].

*Deltaproteobacteria* were detected in MW-B, M16-9-B, M17-10-B, Ba19W-B, and B18-43-B libraries, with 8 OTUs (13 clones), 1 OTU (1 clone), 2 OTUs (2 clones), 2 OTUs (4 clones), and 1 OTU (1 clone), respectively. Among them, 7 OTUs (12 clones with T-RFs of 55 bp, 239–240 bp, 487 bp) from MW-B were closely related to *Syntrophorhabdus* sp. TB (AB611035), *Syntrophus* sp. clones from a methane formation anaerobic system degrading long-chain alkanes [32], and to clones from anaerobic reactors degrading toluene [33] and treating oil production water (GQ844327, GQ844355). *Syntrophorhabdus* is able to degrade benzoate in syntrophic association with a hydrogenotrophic methanogen. The single OTU (1 clone, 241 bp T-RF) from M16-9-B showed 97–98% similarity to *Geobacter* species, which can carry out dechlorination with metal electron acceptors as well as nitrate [34], [35]. One clone from M17-10-B was closely related to a clone from an anaerobic reactor treating oil production water [36]. In contrast, *Deltaproteobacteria* OTUs from Ba19W-B and B18-43-B (T-RFs of 80–81 bp, 483 bp) were closely related to sulfate-reducing *Desulfovibrio* species and sulfur-reducing *Desulfuromonas* species (Fig. 6).

*Epsilonproteobacteria* were retrieved from MW-B, M16-9-B, M17-10-B, Ba19W-B and B18-43-B libraries, with 1 OTU (24 clones, 13.9% of the total clones), 4 OTUs (82 clones, 89.1%), 2 OTUs (102 clones, 56.7%), 1 OTU (48 clones, 61.5%), and 2 OTUs (127 clones, 96.9%), respectively. Among them, *Arcobacter* relatives were dominant, although their diversity was not high in each bacterial library. The *Arcobacter* relatives included 24 clones from MW-B, 95 clones from M17-10-B, 49 clones from M16-9-B, 48 clones from Ba19W-B, and 119 clones from B18-43-B with sequence similarities from 92% to 99%. Some of the clones were also related (99% sequence similarity) to clone DQB-T18 (GQ415365) obtained from an oil reservoir, clone BP-B88 (GQ844394) obtained from petroleum-contaminated groundwater [37], and clones from anaerobic wastewater treatment reactors [36]. Some *Arcobacter* species are able to reduce nitrate and oxidize sulfide [38]–[42]. In addition, OTU M17-10-B13 (7 clones) was closely related to *Sulfuricurvum kujiense*, a sulfur-oxidizing bacterium able to grow on crude oil under anaerobic conditions with nitrate as electron acceptor [43]. OTU M16-9-B02 (32 clones) and OTU B18-43-B01 (8 clones) were closely related to the sulfur-reducing, nitrite-oxidizing bacterium *Sulfurospirillum deleyianum* [44].

The clones from blocks MGL and Ba19 belonging to phyla other than *Proteobacteria* were mainly assigned to uncultured environmental clones retrieved from diverse environments including oilfields, anaerobic reactors, subsurface sediment, petroleum reservoirs, hot springs, lakes, natural gas storage, and sludge (Fig. 5, Fig. 6).

Based on the information from the clone libraries, T-RFLP analysis offered additional information about the bacterial community. Bacteria with T-RFs typically arising from *Alphaproteobacteria* and *Epsilonproteobacteria* were detected in the MGL block. The relative abundances of T-RFs classified as *Alphaproteobacteria* was 23.31% in injection water MW and from 7.47% to 42.72% in the production waters, except M16-9 in which *Epsilonproteobacteria* was predominant. The T-RFs corresponding to *Epsilonproteobacteria* dominated the profiles from MW and five production wells (except M14-8) with a relative abundance of 17.74% to 92.41%. These T-RFs represented bacteria closely related to *Arcobacte*r, *Sulfurospirillum*, and *Sulfuricurvum* relatives. *Gammaproteobacteria* T-RFs corresponding to *Pseudomonas/Acinetobacter* relatives accounted for 8.46% in MW and from 12.74% to 37.23% in three production waters M16-11, M14-8 and M17-10. In contrast to the ubiquity of these bacteria, there were well-specific bacteria existing in large abundance in different wells. The T-RFs of 467 bp and 470 bp (possible *Thauera* relatives) and T-RFs of 522 bp and 524 bp (possible species in *Firmicutes* and *Bacteroidetes* as well as OP11) were unique to wells M14-8 and M15-10. A 55 bp T-RF with an abundance of 10.45% was special for oil well M15-10. Oil well M-J1 was distinguished by the existence of T-RFs of 120 bp (*Denitratisoma* relatives), 90 bp (*Cloacibacterium* relatives), and 115 bp (*Desulfotomaculum* relatives) and the lack of T-RFs of *Pseudomonas/Acinetobacter* relatives and some T-RFs existing in most of the other oil wells. M16-9 and M16-11 were characterized by the low abundance of *Pseudomonas/Acinetobacter* relatives, and the particular existence of T-RFs of 757 bp and 764–767 bp representing relatives of *Sulfurospirillum* (93% similarity) and *Arcobacter* (92% similarity). In the Ba19 block, the relative abundance of T-RFs classified as *Gammaproteobacteria* was 8.47% in injection water and from 50.14% to 99.13% in production waters except for B18-43. *Epsilonproteobacteria* T-RFs representing close relatives of *Sulfurospirillum* and *Arcobacte*r dominated in Ba19W and B18-43 samples, with a relative abundance of 49.06% and 84.93%, respectively. *Alphaproteobacteria* T-RFs were dominant in B18-52 well, with the relative species similar to those in the MGL block. In spite of these common bacteria, there were specific T-RFs existing in large abundance in specific production wells. T-RF of 762 bp was found specifically in B18-35. T-RFs of 467 bp and 470 bp, classified in *Betaproteobacteria/Firmicutes/Bacteroidetes*, were detected in Ba19W and B18-43.

#### Archaeal communities in the two blocks

OTUs assigned to *Euryarchaeota* were dominant in all seven libraries, and most of them were methanogen relatives (Table 3). MGL libraries contained nine genera of methanogens including acetoclastic, methyloclastic, and hydrogenoclastic organisms (Table 3, Fig. S3). However, different samples were dominated by different archaea. *Methanosaeta, Methanolinea*, *Methanomethylovorans*, and *Methanolobus* relatives dominated in MW-A, accounting for 25.3%, 39.1%, 20.7%, and 6.9% of the total archaeal clones, respectively. *Methanosaeta* (43.7%), *Methanocella* (25.6%), and *Methanolinea* (12.6%) relatives dominated in M16-9-A, and *Methanocella* (14.5%), *Methanolinea* (61.8%), and *Methanobacterium* (10.5%) dominated in M17-10-A. *Thermoprotei* relatives (9 clones) were only detected in M16-9-A, closely related to clone WIP_20m_1F_A (EF 420188) from an oil sands tailing pond. OTU M16-9-A07 (5 clones) could not be assigned to any phylum but was closely related to clone mrR2.09 (DQ310430) from the Arctic Ocean. In addition, *Methanoculleus* relatives were only detected in production water, and *Methanospirillum* relatives were only detected in injection water.

Compared to the MGL block, archaeal diversity was much lower in the Ba19 block. Only four methanogenic genera were detected, namely *Methanosaeta*, *Methanosarcina*, *Methanomethylovorans*, and *Methanothermobacter*. *Methanothermobacter* relatives were predominant in all of the Ba19 block samples, accounting for 30.2%, 86.7%, 88.5%, and 62.1% in Ba19W-A, B18-43-A, B18-44-A, and B18-45-A respectively. *Methanomethylovorans* (64.0%) relatives were detected in Ba19W-A. Relatives of *Methanosaeta* and *Methanomethylovorans* each accounted for 12.6% of clones in B18-45-A (Fig. S4). OTUs assigned to *Archaeoglobales* were detected in Ba19W-A, B18-44-A, and B18-45-A. These OTUs showed high sequence similarity to uncultured clones from thermophilic oil reservoirs and from the Gudao Oilfield [9] and had approximately 93% sequence similarities to sulfate-reducing *Archaeoglobus* species (Fig. S4). One OTU (12 clones) from B18-43-A was assigned to *Thermoprotei* and showed 99% sequence similarity to clone NRA16 (HM041917) obtained from a high-temperature petroleum reservoir (Fig. S4).

According to alignments of T-RFs from the clone libraries, samples from MGL shared the following T-RF sizes: 80 bp, 88–89 bp, 185 bp, 254 bp, 388–389 bp, and 517 bp. These T-RFs corresponded to relatives of *Methanolinea*, *Methanothermobacter/Thermoprotei*, *Methanolobus*, *Methanocella*, *Methanosaeta/Methanoculleus/Methanospirillum*, and an unknown *Archaea*. T-RFs of 681–684 bp corresponding to *Methanomethylovorans* relatives were detected in MW, M17-10, and M-J1, but not in the other four production wells. A peak at 225 bp corresponding to *Methanosaeta* relatives did not appear in M15-10 and M16-11. Peaks at 766 bp and 776 bp corresponding to *Thermoprotei* relatives were detected in M14-8, M15-10, and M17-10, but not in MW or the other three production wells. A peak at 70 bp appeared in MW and other three production wells (Fig. 2b).

Based on T-RFLP profiles, the diversity of the archaeal community in Ba19 block was much smaller than the diversity in the MGL block. Peaks at 88–89 bp, 185 bp, 387 bp, and 388–389 bp respectively corresponding to *Methanothermobacter*/*Thermoprotei*, *Methanosarcina*, *Methanothermobacter* relatives, and *Methanosaeta* were detected in all 8 samples. A peak at 225 bp corresponding to *Methanosaeta* relatives appeared in Ba19W and three other production wells. Peaks at 681–684 bp corresponding to *Methanomethylovorans* relatives were detected in Ba19W, B18-45, and B18-44, but not in the other 5 production wells. Peaks at 761–764 bp corresponding to *Archaeoglobales* relatives were detected in Ba19W and 4 other production wells. Peaks at 766 bp and 776 bp were detected in 2 production wells. Peaks of 286 bp, 301 bp, 344 bp, and 362 bp were detected in most of the samples, but their taxonomic affiliation was unknown.

## Discussion

Recently, microbial communities in oil reservoirs were intensively analyzed using injection and production waters from non-flooded, water-flooded, and chemical-flooded oilfields of high-, medium- and low-temperature [1]. The oilfields were classified as sandstone and chalk block with high- and low-salinity and occurred all over the world, including the North Sea, Russia, Canada, The Netherlands, Norway, China, Japan, and the United States [1]. By culture-independent methods, diverse bacteria as well as archaea were detected in these oil reservoirs. However, because the studies are still too limited, it is difficult to connect the microbial communities and geo-physicochemical parameters of oil reservoirs.

The present study investigated microbial communities in production and injection waters from two blocks (the MGL and Ba19 blocks) of different temperature in the Huabei Oilfield. The study used samples of water from 13 oil production wells and 2 injection wells. It is the first study to examine microbial communities in an oil reservoir with samples from so many oil wells and injection waters. The bacterial and archaeal communities in each block and the effect of injection water were examined. The microbial communities of the two blocks were compared to identify possible factors responsible for microbial community differences.

### Characteristic microbial communities in the MGL block

In the MGL block, bacterial communities in the injection water and six production waters from oil wells were quite different. Compared to the production waters, the injection water had a bacterial community of higher diversity and richness. The difference in environmental conditions between the surface and the subsurface might contribute to the differences in the communities. However, compared to reports by Ren et al. (2011), the ratio of bacteria detected both in injection water and production waters was much higher in the MGL block, indicating the possible influence of the bacterial community in the injection water on the communities in the production waters. Twelve OTUs were shared by libraries MW-B (48 OTUs) and M17-10-B (18 OTUs), and 5 OTUs were shared by libraries MW-B and M16-9-B (14 OTUs) (Fig. S2). In addition, 15 T-RFs from MW (total of 20 dominant T-RFs) were detected in production waters. The bacteria shared by the injection water and one production water were not definitely shared by the injection water and another production water, indicating the ambiguity of the influence of injection water on the composition of bacteria in production waters. For example, only 1 OTU was shared by MW-B, M16-9-B, and M17-10-B, whereas 12 were shared by MW-B and M17-10-B and 5 OTUs were shared by MW-B and M16-9-B (Fig. S2). The results indicated that the bacterial community in injection water might be only one of the factors affecting the community in oil wells, and the effect of injection water was not identical for different oil wells.

In addition to the bacterial community differences between the injection water and production waters, the differences in the bacterial communities among the six production waters were also substantial. Only 1 OTU was shared by M17-10-B (20 OTUs) and M16-9-B (14 OTUs) (Fig. S2). There were 11 to 16 T-RFs detected in production waters, but only 4 T-RFs were shared by all 6 production wells and 2 T-RFs by 5 production wells. Five T-RFs were shared by only 2 production wells, and 6 T-RFs were detected in only one well. The low similarity in bacteria communities of two nearby production wells was also observed in the Daqing [10] and Shengli oilfields [9], demonstrating that the heterogeneity among production wells in the same block flooded with the same injection water was not unique to the MGL block. As the salinity, composition of cations and anions, pH, and crude oil composition among oil wells was similar, bacterial community heterogeneity among production wells may arise from differences in geology characteristics, oil layers, or connectedness of injection and production wells. The differences of the oil layers (including the main and sub-layers) among the oil wells in the MGL block were significant, which could be one of the primary factors affecting microbial community composition.

Although the bacterial community structure among production waters was quite different, *Alphaproteobacteria* and *Epsilonproteobacteria* dominated most of the production waters. The *Alphaproteobacteria* included close relatives of the genera *Novosphingobium*, *Brevundimonas*, *Caulobacter*, *Sphingomonas*, *Sphingobium*, and *Rodobacter*, most of which are able to reduce nitrate and degrade aromatic compounds and are often detected in oil reservoirs [5], [9], [10], [45]. The *Epislonproteobacteria* had the highest relative abundance and included relatives of the genera *Sulfurospirillum* and *Arcobacter*, both of which are connected to the cycling, oxidization, and reduction of sulfur and nitrogen. Some species in *Arcobacter* are able to reduce nitrate and oxidize sulfide, while some species in *Sulfurospirillum* are able to reduce sulfur and oxidize nitrite. Their co-occurrence in relatively high ratios in oil well communities suggests the organisms play important roles in sulfur and nitrogen cycling in the oil reservoir. *Arcobacter* species are dominant in some oil reservoirs [4], [46]. Though *Arcobacter* relatives were detected as dominant bacteria in the MGL block, their function *in situ* in oil wells remains unclear, because few strains have been isolated from oil reservoirs and functionally characterized.

In addition to the common dominance *Alphaproteobacteria* and *Epsilonproteobacteria* species in most of the oil wells, each production water had its specific dominant species, revealing the difference in the bacterial community in each well. Therefore, the study of only one or two oil wells is insufficient to understand the bacterial community of an entire oil reservoir, or even a block.

Compared to the bacterial communities, the archaeal communities dominated by methanogens were relatively consistent in the MGL block. Community differences among the injection water and production waters from different oil wells were not so substantial. Most of the clones as well as T-RFs detected in production waters were also found in the injection water MW (Fig. 2b, Fig. S2). This contradicted the report of Ren et al [9], in which archaeal communities were significantly different among injection water and oil wells. The MGL block, the Ba19 block, as well as other blocks we investigated in the Xinjiang and Daqing Oilfields (data not shown) showed block-specific archaeal communities, suggesting that stability of archaeal communities among production wells in the same block is common. In other words, archaeal communities are not as sensitive as bacterial communities to changes in environmental conditions. The results are consistent with reports that methanogens are probably indigenous members of oil reservoir microflora [47], [48] and their community composition is determined mainly by the oil reservoir itself.

Methanogens were dominant in all samples. The relatives of the genera *Methanolinea*, *Methanothermobacter*, *Methanocella*, and *Methanosaeta* were dominant in all samples, while relatives of *Methanomethylovorans* were only detected in M17-10 and M-J1 as well as injection water MW (Fig. 2b). The methanogens detected in the present study were also detected in other oil reservoirs. However, the diversity of methanogens in the MGL block was higher than other moderate and low temperature oil reservoirs [4], [12]. In the MGL block, hydrogenotrophilc methanogens were the predominant in methanogens, suggesting that carbon dioxide reduction to methane might be more prevalent than acetoclastic methanogenesis in this moderate-temperature oil reservoir. Relatives classified as *Thermoprotei* were only detected in oil wells such as M14-8 and M15-10 but not in injection water, indicating they are possibly indigenous to the oil reservoir.

The diversity of bacteria and archaea in the production waters in the MGL block indicated a relatively high activity of microbial communities responsible for crude oil degradation, especially degradation of saturates and aromatics because the ratio of saturates and aromatics in crude oil was substantially lower in the MGL block compared to the nearby Ba19 block. The microbial community in production waters from the MGL block showed excellent emulsification and crude oil degradation capacity in lab experiments when production water was used as medium without addition of extra nutrients (data not shown).

### Characteristic microbial communities in the Ba19 block

As in the MGL block, bacterial diversity was higher in injection water than in seven production waters in the Ba19 block. However, among 13 OTUs in Ba19W-B, only three were detected in B18-43B, and none of them was detected in B18-44B and B18-45B. Among 12 T-RFs detected in Ba19W, 6 were detected in some of the production waters; only one T-RF was detected in all 7 production waters. It indicated that the bacterial community in the injection water did not show an obvious influence on the bacterial communities in production wells, similar to findings with a thermophilic block in Shengli Oilfield reported by Ren et al. [9].

In the present study, the bacterial community revealed by clone library analysis was relatively simple in production waters. The low number of T-RFs detected in production waters and the strong dominance of a few T-RFs indicated a relatively simple community in the Ba19 block, which was high-temperature compared to the moderate-temperature MGL block.

Both clone library analysis and T-RFLP analysis revealed the predominance of relatives in the genera *Pseudomonas* and *Acinetobacter* of the *Gammaproteobacteria* in the production waters in the Ba19 block. The relative abundance of T-RFs of the *Pseudomonas* and *Acinetobacter* relatives was more than 50% in production waters except B18-43. In addition to the predominant *Pseudomonas* and *Acinetobacter* relatives, species related to *Sulfurospirillum* and *Arcobacter* in the *Epsilonproteobacteria* were detected in production waters. B18-52 and B18-43 were unusual compared to other production waters. *Pseudomonas* and *Acinetobacter* relatives and species in *Alphaproteobacteria* were dominant in B18-52. B18-43 was unique in being dominated by species related to *Sulfurospirillum* and *Arcobacter*. These results show that the bacterial community of an oil reservoir or a block can not be represented by only one or two production wells.

Though there are reports of thermophilic bacteria dominating high-temperature reservoirs [2], [5], [7], we found a different pattern. Regardless of the *in situ* temperature, *Pseudomonas* and *Acinetobacter* relatives are commonly found as dominant bacteria in oil reservoirs [4], [6], [9], [10], [12]. However, the predominance of *Pseudomonas* and *Acinetobacter* relatives in the Ba19 block, which was high-temperature, was unexpected. There are researches reporting the predominance of *Pseudomons* in the high temperature oil wells, however, in these cases only one oil well was investigated [6], [9]. The predominance of *Pseudomonas* and *Acinetobacter* in one block with such many oil wells studied was reported at the first time in the present study. Similar results were obtained when we studied another thermophilic block Zan3 in Shengli Oilfield (data not shown).It might be common that *Pseudomonas* and *Acinetobacter* relatives predominated in high temperature oil wells which were water flooded for a long time. Though pure cultured *Pseudomonas* and *Acinetobacter* species are prefer to grow in moderate temperature range, they might be able to survive and even grow in thermophilic condition, compared to other species in injection water, which lead to the big increase of the relative abundances of *Pseudomonas* and *Acinetobacter* relatives and lower microorganism diversity. In addition to *Pseudomonas* and *Acinetobacter* relatives, most of the remaining bacteria detected in production waters in the Ba19 block were mesophilic bacteria. It could be deduced that bacterial communities in the production waters in the Ba19 block were mainly introduced from injection water during oil production by long-term water flooding.

Thermophilic *Methanothermobacter* and *Methanosaeta* relatives were predominant methanogens in production waters in the Ba19 block. *Methanomethylovorans* relatives were detected in high abundance in injection water Ba19W, but they were not detected in most of the other production waters except for B18-45, indicating the oil reservoir environment might be unsuitable for growth. In addition to methanogens, *Thermoprotei* species were detected in two production waters, though their function is still unclear. Sulfate-reducing *Archaeoglobales* species were found not only in injection water but also in two production waters. *Thermoprotei* and *Archaeoglobales* relatives are commonly found in high-temperature oil reservoirs [3], [5], [9], [49].

Both bacterial and archaeal communities in the Ba19 block were relatively simple. The dominant bacteria and archaea in different production waters were basically identical, suggesting a block-specific character, which might be due to the similar oil layers among production wells. However, compared to other high-temperature oil reservoirs, the diversity of the microbial community in block Ba19 was relatively low. The reason for the predominance of one or two bacteria genera merits further study.

### Comparison of microbial communities between the two blocks

Although the two blocks had different geological characteristics, bacterial and archaeal communities in the injection water were more similar between blocks compared to communities from production well water within blocks. In other words, the injection water communities from the MGL and Ba19 blocks shared more common bacterial phyla and methanogens. One of the possible reasons is the similar origin of the injection water. The injection water was originally from surface water at two geographically close locations, which should harbor similar microbial communities.

In addition to the similarity of the microbial communities in the two injection waters, bacteria in similar phyla were detected in the production water (representing the oil well conditions) of both blocks but were substantially different at the class level. The predominant bacteria detected in MGL production waters were *Alphaproteobacteria* and *Epsilonproteobacteria*, while *Gammaproteobacteria* and *Epsilonproteobacteria* were dominant in the Ba19 production waters. At the genus level, substantial differences in bacterial communities were also detected between the two blocks. The situation was similar for archaeal communities. More thermophilic methanogens related to *Methanothermobacter* (as the predominant archaea) and *Methanomethylovorans* were detected in Ba19 production waters compared with *Methanosaeta, Methanocella, and Methanolinea* relatives in MGL production waters. It is interesting that although the MGL sandstone block and the Ba19 fault block were within 50 km of each other, their bacterial and archaeal communities in production water were substantially different. One reason could be the different geological characteristics of the two blocks. MGL is younger in sedimentation and shallower in depth than the Ba19 block, resulting in lower temperature, lower reservoir pressure, higher reservoir porosity, and different oil characteristics. As a consequence, the MGL block harbors a more complex and diverse microbial community than the Ba19 block. In spite of the obvious differences in diversity and predominant microbes between the two blocks, the two blocks shared some taxa, at least at the genus level. This was supported by the clustering pattern of the bacterial T-RFs. The separate clustering of the archaeal communities between the two blocks may be due to the different injection water archaeal communities and the significantly different oil well temperatures.

In the same Huabei Oilfield, Li *et al.* reported bacterial and archaeal communities comprised of *Gammaproteobacteria* (85.7%), *Thermotogales* (6.8%), *Epsilonproteobacteria* (2.4%), low-G+C Gram-positive (2.1%), high-G+C Gram-positive, *Betaproteobacteria* (<1.0%), *Nitrospira* (<1.0%) [6], and archaea closely related to *Methanothermobacter, Methanobacterium, Methanococcus, Methanocaldococcus, Methanocorpusculum, Methanoculleus, Methanoplanus, Methanomicrobium, Methanocalculus, Methanosarcina, Methanosaeta* [11]. It is unclear which block Li et al. investigated, and how far the block was from the present two blocks. However, the *in situ* temperature (75°C), the depth (1500–1700 m), and salinity (16,622 mg/l) of their block show that it was different from the present ones.

MEOR techniques should be developed for different blocks due to the different microbial communities that occur among blocks. Ubiquitous microbes should be the targets of stimulation because it is not feasible to accommodate every bacterial species. Due to the environmental complexity of oil reservoirs, it would be normal that differences would appear in the stimulation efficiencies of different wells due to the differences among microbial communities. Future studies that clarify the correlation between microbial communities and characteristics of oil reservoirs may reveal factors influencing microbial communities, guiding the development and application of MEOR techniques.

## Supporting Information

Figure S1 **Rarefaction curves of bacterial and archaeal 16S rRNA gene clone libraries.**
(DOC)

Figure S2 **Venn diagram showing the distribution of bacterial and archaeal OTUs in the MGL and Ba19 blocks.**
(DOC)

Figure S3 **Phylogenetic tree showing the genetic relationships among archaeal clones from the MGL block.**
(DOC)

Figure S4 **Phylogenetic tree showing the genetic relationships among archaeal clones from the Ba19 block.**
(DOC)
