# Supplementary material for: Microbial Communities in Long-Term, Water-Flooded Petroleum Reservoirs with Different in situ Temperatures in the Huabei Oilfield, China
Source: PLoS One. 2012 Mar 14;7(3):e33535. doi: 10.1371/journal.pone.0033535 (PMC3303836; doi:10.1371/journal.pone.0033535)
Supplement: Figure S1 — Rarefaction curves of bacterial and archaeal 16S rRNA gene clone libraries. (DOC) [file pone.0033535.s001.doc]

Figure S1 Rarefaction curves of bacterial and archaeal 16S rRNA gene clone libraries. (a) the MGL block and (b, c) the Ba19 block. OTUs were defined at a 97% sequence similarity level. Curves were generated by 10,000 iterations of random sampling. Error bars represented 95% confidence intervals (CI) of the observed OTUs

a

b
