# Supplementary material for: Microbial Communities in Long-Term, Water-Flooded Petroleum Reservoirs with Different in situ Temperatures in the Huabei Oilfield, China
Source: PLoS One. 2012 Mar 14;7(3):e33535. doi: 10.1371/journal.pone.0033535 (PMC3303836; doi:10.1371/journal.pone.0033535)
Supplement: Figure S2 — Venn diagram showing the distribution of bacterial and archaeal OTUs in the MGL and Ba19 blocks. (DOC) [file pone.0033535.s002.doc]

Figure S2 Venn diagram showing the distribution of bacterial and archaeal OTUs in the MGL and Ba19 blocks.
