# Supplementary material for: Microbial Communities in Long-Term, Water-Flooded Petroleum Reservoirs with Different in situ Temperatures in the Huabei Oilfield, China
Source: PLoS One. 2012 Mar 14;7(3):e33535. doi: 10.1371/journal.pone.0033535 (PMC3303836; doi:10.1371/journal.pone.0033535)
Supplement: Figure S3 — Phylogenetic tree showing the genetic relationships among archaeal clones from the MGL block. (DOC) [file pone.0033535.s003.doc]

Figure S3 Phylogenetic tree showing the genetic relationships among archaeal clones from the MGL block. The tree was constructed by the Neighbor-Joining method using partial sequences of 16S rRNA gene. Numbers of clones with identical sequences are shown in parentheses. The bar represents two substitutions per 100 nucleotide positions. Bootstrap probabilities >70% are indicated at the branch nodes. The DDBJ/EMBL/GenBank accession numbers for reference strains and clones obtained in this study are shown in parentheses.
